# Medical Aspects of Sterility in Women

**Published:** 1919-10-18

**Authors:** 


### A BIRTH-RATE QUESTION.

### Medical Aspects of Sterility in Women.

It is perhaps one of the beneficial changes which the war has produced in this country that there is a noticeable decrease in the formerly obvious tendency for young married people to shirk the responsibility of parentage. The instinct of motherhood in women and that of fatherhood in men would appear to have been strongly stimulated, and, needless to remark, such development deserves the fullest encouragement from the medical profession, and in this connection, taking one aspect at least, the doctor cannot do better work than to spread teaching directed to prevention of sterility in women and to practice to the utmost the means which may bring about its cure. Recently, in a lecture delivered in the Fellowship of Medicine Post-Graduate Clinic, A. E. Giles, M.D., B.Sc., F.R.C.S., senior surgeon to the Chelsea Hospital for Women, gave the profession a most useful review and reminder of the situation as it lies before them, and from his words we venture to abstract certain points, which, though perhaps well known to the expert, appear to us of great value to the general practitioner, who generally meets these cases in the first instance.

### Hopeless Cases.

Dr. Giles disposes at the outset of the hopeless type of sterility, giving it this place in his lecture as much from the fact that we must be very sure of our ground before passing the sentence of barrenness on a woman, as for the absolute impossibility of successful medical assistance. He classifies the incurable cases under three headings—namely: (1) Certain malformations, such as under-development of uterus, atresia, etc.; (2) new growths such as bilateral ovarian tumours, sarçoma, and carcinoma of the uterus, and some types of uterine filroid; (3) inflammatory conditions whereby the patency of the Fallopian tubes is irretrievably damaged. The chief requirement is here diagnostic skill, and it is both more practical and profitable to turn to the more hopeful side of the subject.

### Preventable Causes.

The preventable causes of sterility fall under two categories, age and inflammatory conditions. Theoretically a woman may conceive at any time during the period of sexual maturity. In practice it is found that there is an age—twenty to twenty-four—of maximum fertility, and that from the age of twenty-five onwards there is a steady increase in the percentage of sterile marriages. These facts, first noted by Matthews Duncan, have never been disproved.

Obviously a large amount of the sterility which has existed might have been prevented had the woman married in the early twenties instead of the early thirties. Therefore should earlier marriages be encouraged from both natural and domestic points of view. Modern circumstance has, we are well aware, been all against such an ideal, but, as Dr. Giles adds:

> A corollary to these considerations is that when a marriage is unfruitful, *early investigation* into the case should be made; the condition may be easily remediable; but the outlook is much better if treatment is undertaken at the age of twenty-five than if it is postponed to the age of thirty-five.

Inflammatory conditions are probably the next greatest cause of sterility. Figures given in the lecture show that in these cases 10 per cent. had endometris or vaginitis, 11 per cent. had tubal disease, and over 4 per cent. gave a history of peritonitis. It is suggested that untreated early attacks of appendicitis were the root cause in an appreciable number of cases, the associated peritonitis involving the delicate tubal ostia, permanently sealing them up, and, from this, the moral is drawn that young women who develop appendicular trouble should be operated upon soon to avert possible tubal involvement. The sequence and effects of gonorrhœal infection are only too well known, and go to swell the amount of responsibility held by local inflammations. Quoting from Dr. Giles's book recently published by the Oxford Press:

> In all there were 111 cases out of 530, or nearly 21 per cent.—in other words, one case in every five, in which sterility was attributable to pelvic inflammation. . . . We found in Chapter II. that at least 10 per cent. of marriages are unfruitful; and now we see that in one-fifth of that 10 per cent. the cause may be attributed to pelvic inflammation. Thus two women out of every hundred married are sterile on this account. If we assume that on the average three children are born from each fruitful marriage, then six children are lost to the future population for every hundred women married. The number of marriages in the United Kingdom every year is about 333,500. Consequently, every year the chance of some 20,000 children being added to the future population is lost, *owing to preventable causes.*

### The Curable Causes.

The curable causes of sterility may be conveniently classified into two divisions—*i.e.*: (1) Conditions which actually interfere with intercourse, and (2) conditions which interfere with ascent of the spermatozoa. Of the first it is well known that dyspareunia may be caused by some of the simplest clinical conditions, the great majority of which can be rectified by relatively simple treatment. The difficulty lies mainly in overcoming a patient's reticence and achieving the full sympathy which is essential to success in these matters. Vaginismus, a functional condition, is generally amenable to dilatation under anæsthesia and judicious medical advice. Another type of obstacle is found in the presence of pelvic tumours encroaching on the vagina, and care must be taken to differentiate between those which may be readily removed, and those which are from the first hopeless since the process of removal places conception finally out of the question.

With regard to interference with the spermatozoa,

**A Birth-rate Question**—(*continued*).

Dr. Giles reminds us of one or two points which are not always appreciated. There is the bio-chemical type—associated with toxic, especially acid, discharges as accompany erosion, endometritis, and many forms of vaginitis, the treatment of such conditions being straightforward but requiring perhaps more thoroughness than is often applied. Mechanical interference is occasioned by mal-position of the uterus. Acute anteflexion, with stenosis of the external os, is the commonest, and excellent results may be obtained by dilatation and simultaneous straightening of the uterine axis; but, as the author explains:

"It is important that the dilatation be carried out to a sufficient degree; and it is also important, for the credit of the treatment, that cases be carefully selected. If every case of sterility that is encountered is treated by dilatation a considerable proportion of failures will inevitably result. We may go further and say that this treatment should not be advised unless the medical attendant is satisfied that the husband can be absolved from responsibility for sterility."

In retroversion and retroflexion improvement may follow the use of a Hodge pessary, and the expedient may be given preliminary trial, but it must be remembered that these instruments tend to produce the acid discharge already noted. A hysteropexy properly carried out is often far better. The whole is but a very brief review of a very wide and important subject, but it does, as is intended, throw into relief the practical and important fact that, provided the cause of sterility be carefully investigated in every case, we may, more frequently than is generally realised, have the satisfaction of bringing to many parents the attainment of their dearest wish.

